# Sirolimus-Associated Pruritus: Case Report and Review

**DOI:** 10.7759/cureus.1398

**Published:** 2017-06-27

**Authors:** Joyce Y Cheng, Philip R Cohen

**Affiliations:** 1 Department of Internal Medicine, University of California, San Diego; 2 Department of Dermatology, University of California, San Diego

**Keywords:** heart, itch, pruritus, sirolimus, transplant

## Abstract

Sirolimus is an immunosuppressant drug used to prevent organ rejection in transplant patients. We describe a man with sirolimus-associated pruritus and review the features of this adverse event in other individuals receiving this drug. The patient was a 67-year-old heart transplant recipient receiving sirolimus as part of his immunosuppressive regimen. He developed severe pruritus over the distal extremities, face, and earlobes six months after starting the drug. The symptoms became progressively worse as he continued to receive this medication. Temporary elimination of the drug resulted in cessation of his itching. Subsequently, sirolimus was discontinued and everolimus was started; this provided temporary relief of his pruritus. PubMed was used to review the following terms: “sirolimus”, “itch”, and “pruritus.” Relevant papers and their references were reviewed. We are aware of only one other patient in whom pruritus necessitated cessation of treatment with sirolimus. Systemic pruritus is a rare adverse event associated with sirolimus. It can occur in both heart and liver transplant patients, beginning several months after transplant, and typically persists. Dose reduction may improve symptoms. Discontinuation of the medication or use of alternative immunosuppressants may be necessary for complete symptom relief.

## Introduction

Sirolimus is an mammalian target of rapamycin (mTOR) inhibitor that suppresses T-cell proliferation [1]. It has become a mainstay in immunosuppressive therapy for solid organ transplant patients. We describe a 67-year-old man who received a heart transplant and subsequently developed sirolimus-associated pruritus.

## Case presentation

A 62-year-old man received a heart transplant secondary to nonischemic dilated cardiomyopathy in February 2013. His posttransplant immunosuppressive regimen consisted of mycophenolate mofetil, prednisone, and tacrolimus. Mycophenolate mofetil was stopped in April 2013 due to increased development of skin cancers. Sirolimus was started in October 2013, with prednisone and tacrolimus, for its antiproliferative effects on cutaneous squamous cell carcinomas. For an elective surgery in December 2013, the patient’s sirolimus was temporarily replaced with azathioprine, while prednisone and tacrolimus were continued. Sirolimus was resumed in March 2014 alongside azathioprine and tacrolimus, while prednisone was stopped. Azathioprine was stopped in August 2014. In September 2014, the patient began to complain of itching.

The patient’s pruritus was predominantly localized to his distal extremities, face, preauricular area and postauricular area. It occurred at least three to four days every week for about three years. It was not relieved by oral antihistamines, topical corticosteroids, or topical antipruritic lotions containing camphor and menthol.

The severity of the pruritus prompted the patient to alternate discontinuation of his immunosuppressant drugs. When he eliminated sirolimus, all symptoms resolved. When he reinitiated the medication, the pruritus recurred.

His transplant physicians decreased the daily dosage of sirolimus. The pruritus persisted. Subsequently, they substituted everolimus for the sirolimus in October 2016. He remained symptom-free for five months. However, his pruritus recurred; it significantly improved but did not completely resolve when the dose of everolimus was decreased.

## Discussion

Immunosuppressant drugs used following solid organ transplant include mTOR inhibitors such as everolimus and sirolimus, calcineurin inhibitors such as cyclosporine and tacrolimus, mycophenolate mofetil, and prednisone [2]. The immunosuppressive regimen is chosen based on the organ transplanted and the experience of the physicians managing the patient [2]. Our patient’s immunosuppressive regimen initially included mycophenolate mofetil, prednisone, and tacrolimus.

mTOR inhibitors act by blocking the mammalian target of rapamycin complex, leading to immunosuppression and antiproliferative effects [2]. Common side effects of sirolimus include hyperlipidemia, leukopenia, peripheral lymphedema, and thrombocytopenia [3]. Cutaneous adverse effects are uncommon in patients receiving sirolimus. They include acneiform dermatitis, folliculitis, onychopathy, oral ulcers, rash, and stomatitis [3-4].

Systemic pruritus associated with sirolimus is rare. To the best of our knowledge, this has only previously been described in a 56-year-old woman who received a liver transplant. After failing a number of immunosuppressive therapies due to side effects, the patient was switched to sirolimus. Three months into treatment with sirolimus, she developed generalized pruritus and an ulcerating maculopapular rash involving her trunk, arms, and legs [5]. Similar to in our patient, her pruritus was severe and drug cessation was required. Her symptoms resolved shortly thereafter.

Other mTOR inhibitors have been used for immunosuppression following solid organ transplantation; they have also been associated with side effects. Everolimus is a derivative of sirolimus with a similar mechanism of action. It has been associated with a significantly increased risk of oral ulcers, skin rash, and stomatitis [5-6]. Topical sirolimus has been used in the treatment of facial angiofibromas in tuberous sclerosis with no observed systemic effects; however, local irritation is the most commonly reported side effect [7]. In our patient, substituting everolimus for sirolimus initially resulted in complete resolution of symptoms. However, the pruritus eventually recurred and improved when the dose of everolimus was lowered.

## Conclusions

Sirolimus-associated pruritus is a rare cutaneous adverse event associated with systemic administration of the drug. Although uncommonly described, it appears to be a drug-limiting side effect requiring substitution of an alternative immunosuppressant. The pathogenesis of sirolimus-induced pruritus remains to be established. Our patient initially had complete resolution of his symptoms when switched to another mTOR inhibitor, everolimus. However, the pruritus eventually recurred. Hence, an alternative class of immunosuppressive medication may be indicated in a patient who develops severe pruritus in response to an mTOR inhibitor.
